# Spectral photoacoustic imaging of age-related reproductive tract collagen changes in a mouse model of prolapse

**DOI:** 10.1016/j.pacs.2026.100810

**Published:** 2026-02-12

**Authors:** Andrew C. Markel, Viraj Puri, Mari J.E. Domingo, Kristin S. Miller, Carolyn L. Bayer

**Affiliations:** a Department of Biomedical Engineering, Tulane University, 500 Lindy Boggs Center, New Orleans, LA 70118, USA; b Department of Bioengineering and Mechanical Engineering, University of Texas at Dallas, 800 W. Campbell Road, Richardson, TX 75080, USA; c Department of Obstetrics and Gynecology, University of Texas Southwestern Medical Center, 5323 Harry Hines Blvd, Dallas, TX 75390, USA

**Keywords:** Pelvic organ prolapse, Reproductive tract, Collagen, Aging, Photoacoustic imaging, Spectral unmixing, NIR-I

## Abstract

Monitoring collagen may help physicians assess female reproductive health, since altered collagen with age is an established risk factor for female reproductive disorders including pelvic organ prolapse. Currently, quantifying collagen in soft tissues requires a biopsy, exposing patients to unnecessary risk with unavoidable site bias. We are developing ultrasound-guided spectral photoacoustic imaging methods to estimate reproductive tract collagen in a Fibulin-5 knock out mouse model of prolapse. For mice younger than one year old, collagen concentration in the external cervical os correlated strongly with age in normal mice (R^2^ = 0.701, p = 9.54e-3) but not in prolapsed mice (R^2^ = 0.340, p = 1.29e-1). Mice aged 4–8 months suffering from prolapse had significantly less collagen in the external cervical os (p = 2.99e-2). Spectral photoacoustic imaging is a potential non-invasive clinical tool for monitoring changes in reproductive tract collagen.

## 1 Introduction

Collagen is a critical component in the female reproductive tract. Collagen confers the strength and stability necessary for normal reproductive function, while collagen remodeling ensures homeostasis during physiological events such as pregnancy, childbirth, and postpartum recovery [1], [2]. Changes in collagen metabolism can negatively impact female reproductive health, resulting in conditions such as pelvic organ prolapse (POP) [3] and preterm birth [4]. Aging is a risk factor for both POP [5] and preterm birth [6], so regular monitoring of reproductive collagen may help physicians to evaluate women for risk of collagen-related reproductive disorders.

Better tools are needed to longitudinally monitor female reproductive collagen changes in the clinic. Current methods for quantifying collagen in soft tissues require a biopsy sample to be taken from the patient [7]. Biopsies are not ideal for routine monitoring because they are invasive, exposing patients to unnecessary risk, and spatially limited, providing information about only the small area of tissue that was extracted. New tools for longitudinal monitoring of collagen in the female reproductive tract must be non-invasive and repeatable, allowing for larger areas of tissue to be assessed multiple times.

Ultrasound-guided spectral photoacoustic (sPA) imaging has the potential to non-invasively and repeatably assess reproductive tract collagen. Ultrasound imaging is a useful tool for assessing anatomical structures in the pelvic floor (which includes the reproductive tract) [8], but it lacks the molecular imaging capabilities required to quantify changes in collagen. sPA is a molecular imaging complement to ultrasound that has been applied to study collagen content in several organ models, including muscle [9], kidney [10], bone [11] and cartilage [12]. Researchers studying cervical remodeling were able to differentiate between different microstructural states of the cervix throughout pregnancy by quantifying the collagen to water ratio [13], [14], [15] using spectral photoacoustic imaging; however, these studies focused only on cervical tissue and did not characterize vaginal or uterine tissues. Furthermore, previous photoacoustic imaging research on cervical remodeling used the second near-infrared region of the electromagnetic spectrum (NIR-II, 1000–1700 nm), which is less common in commercial photoacoustic imaging systems and conventional optical parametric oscillator (OPO) lasers compared to the first near-infrared region (NIR-I, 650–1000 nm).

Our goal is to develop sPA methods in NIR-I to assess age-related changes in reproductive tract collagen in an established mouse model of POP. The Fibulin-5 (*Fbln5*) gene is crucial for proper elastic fiber assembly. Mice with *Fbln5* knocked out develop severe prolapse by 6 months of age [16]. Previous research studying this mouse model found significant differences in vaginal [17] and cervical [18] collagen structure and composition in prolapsed tissues but did not directly address how the overall collagen content in the reproductive tract changes with prolapse. Previous research has also demonstrated age-related changes in the vagina [19], cervix [20], and uterus [21] of normal mice, but age-related reproductive collagen changes in prolapsed mice have not yet been established. This animal study is a step towards clinical translation of sPA imaging for longitudinal monitoring of female reproductive health.

## 2 Methods

### 2.1 Animal care

The Institutional Animal Care and Use Committee at Tulane University approved all procedures in this study. Female and male *Fbln5*^+/-^ mice on mixed background (C57BL/6 × 129SvEv) generated all female *Fbln5*^+/-^ and *Fbln5*^-/-^ mice used within this study. A total of sixteen female, nulliparous mice 2–12 months of age were used in this study. Prior to sacrifice, mice were assessed for prolapse using the Mouse Pelvic Organ Prolapse Quantification system, where a grade of 0 corresponds with a healthy animal with no perineal bulge, 1 corresponds with a small but detectable bulge, 2 with a moderate bulge, 3 with a large bulge, and 4 with the vagina coming out [22]. Eight *Fbln5*^+/-^ mice with no perineal bulge (grade 0) and eight *Fbln5*^-/-^ mice that developed a moderate to large perineal bulge (grades 2 and 3) were considered in the study. All sixteen mice aged 2–12 months were used to investigate the relationship between collagen and aging, while a subset of eight mice aged 4–8 months were used in studies investigating the relationship between collagen and prolapse in age-matched tissue samples. The age-matched group included four *Fbln5*^+/-^ mice to serve as the normal group and four *Fbln5*^-/-^ mice to serve as the prolapsed group. Animals were euthanized via CO_2_ asphyxiation and received secondary euthanasia in the form of cervical dislocation.

### 2.2 Tissue preparation

**Fig. 1:**
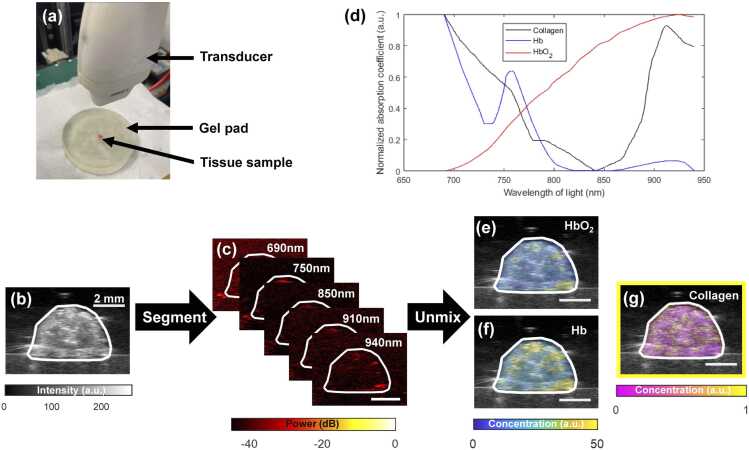
Collagen concentration estimation via spectral unmixing. (a) Normalized absorption spectra of collagen, deoxygenated hemoglobin (Hb), and oxygenated hemoglobin (HbO_2_) used in the spectral unmixing algorithm. (b) Imaging setup. (c) B-mode ultrasound image used to segment the tissue (white boundary). (d) Photoacoustic images acquired at various wavelengths. Unmixed relative concentration maps of (e) HbO_2_, (f) Hb, and (g) collagen.

**Fig. 2:**
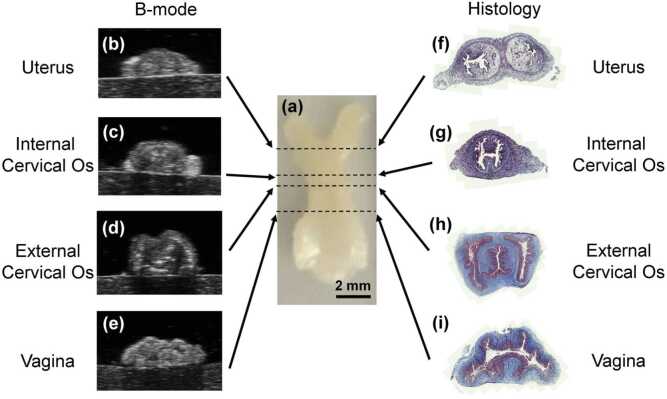
Alignment of imaging analyses in the murine reproductive tract. (a) Example excised reproductive tract tissue sample. Example B-mode ultrasound images of the (b) uterus, (c) internal cervical os, (d) external cervical os, and (e) vagina. Example tissue sections of the (f) uterus, (g) internal cervical os, (h) external cervical os, and (i) vagina stained with Masson’s trichrome.

The reproductive tract was excised as previously described [17], [23]. Briefly, the reproductive tract was isolated by making incisions at the two uterine horns below the ovaries and cutting around the introitus. (Fig. 2(a)). Each intact reproductive tract was soaked in saline solution for at least 30 min to remove most of the blood from the tissue. The tissue was removed from the saline bath, placed on an ultrasound gel pad (Hill Laboratories, Frazer, PA, USA), and covered with ultrasound coupling gel to prepare for imaging (Fig. 1(a)).

### 2.3 Imaging

Co-registered ultrasound (Fig. 1(b)) and photoacoustic images (Fig. 1(c)) of each organ along the reproductive tract (Fig. 2) were acquired using the Vevo 2100 multimodal imaging system and LZ550 transducer (FUJUFILM VisualSonics, Toronto, ON, Canada) integrated with a Phocus HE benchtop OPO laser (Opotek, Carlsbad, CA, USA). Images were co-registered by interleaving ultrasound and photoacoustic image acquisitions using the same probe. Photoacoustic images were acquired at five different wavelengths of light to emphasize various spectral features for the collagen absorption spectrum in NIR-I (Fig. 1(d)). Moving from lowest to highest frequency: we chose to image the maximum collagen absorption in NIR-I at 690 nm, the frequency where our laser’s fluence was highest at 750 nm, the local minimum in the collagen absorption spectrum at approximately 850 nm, the local maximum at approximately 910 nm, and the local minimum at approximately 940 nm.

3D volumes were acquired at each wavelength using a scanning motor with a 0.032 mm step size. To conserve digital storage space while still capturing the important anatomical structures across the reproductive tract, we acquired partial scans of the tissue sample, ensuring that the vagina, cervix, and uterus were included. The organs along the reproductive tract were then identified using the B-mode ultrasound images (Fig. 2(b-e)) and manually segmented in MATLAB. New ROIs were drawn for each frame in the scan.

### 2.4 Spectral unmixing

We used a spectral unmixing algorithm to estimate the relative concentrations of collagen, oxygenated hemoglobin (HbO_2_), and deoxygenated hemoglobin (Hb) in each organ, adapting methods used by Lawrence et al. [24]. While Lawrence et al. assumed that photoacoustic signals were generated solely by Hb and HbO_2_, our algorithm adds collagen as well as blood hemoglobin. Furthermore, Lawrence et al. collected photoacoustic images using three wavelengths of light to unmix the concentrations of two chromophores, whereas we collected five wavelengths to unmix three chromophores. Lastly, the results presented in research by Lawrence et al. focus primarily on measuring blood oxygenation, while our approach focuses primarily on measuring collagen concentration.

Our spectral unmixing algorithm included absorption spectra from hemoglobin in addition to collagen because reproductive tissues are highly vascularized and because blood hemoglobin generates strong photoacoustic signals in NIR-I. Even though we washed most of the blood from our excised tissue samples, trace amounts of blood still influenced photoacoustic image contrast; therefore, Hb and HbO_2_ were still included in the unmixing algorithm. Additionally, including hemoglobin allows our unmixing algorithm to be adopted to *in vivo* imaging without additional modifications.

The molar absorption spectrum of collagen was calculated using the absorption coefficient and mass density from Sekar et al. [25] along with the molar mass of collagen from Kimura and Tanzer [26]. Molar absorption coefficients for oxygenated and deoxygenated hemoglobin were obtained from data compiled by Prahl [27]. Before unmixing the chromophore concentrations, the molar absorption spectrum for each chromophore was scaled from 0 to 1 using min-max normalization (Fig. 1(d)). Hemoglobin absorbs light orders of magnitude more strongly compared to collagen, so spectrum normalization allows the spectral unmixing algorithm to equally weight the signal contributions from collagen [10].

The relative collagen and hemoglobin concentrations (Fig. 1(e-g)) of each co-registered image frame were calculated in MATLAB (MathWorks, Natick, MA, USA). We assumed the Gruneisen parameter and laser fluence were constant, while each chromophore’s concentration varied with space and each chromophore’s light absorption varied with probing light wavelength. We therefore assumed that photoacoustic signal intensity generated by each chromophore was proportional to the product of its spatial concentration and its absorption coefficient at the imaging wavelength. We also assumed the intensity of each pixel in the resulting photoacoustic image to be the sum of the signal intensities from only collagen and hemoglobin in the corresponding area in the tissue.

To ensure that wavelength-to-wavelength variations in energy would not affect our unmixing results, each photoacoustic image was normalized by the light fluence used to acquire it before solving for relative chromophore concentrations. Light fluence for each laser pulse was estimated by diverting a fraction of the light beam to measure its energy, and dividing the measured energy by the area of the beam at the focal point (0.51 cm^2^). The average fluence for each wavelength was calculated by averaging the estimated fluence for all the pulses recorded across the respective 3D scan. From each fluence-normalized 3D scan, the selected frames corresponding to each organ along the reproductive tract were then fed into the spectral unmixing algorithm.

For this preliminary work, we assumed the sample contained only blood and collagen, and forced the sum of the resulting collagen and hemoglobin concentration estimates to equal 1. Any concentration values less than 0 were replaced with a value of 0. The values presented are relative concentrations, thus they are presented in arbitrary units (a.u.). To better visualize the relatively smaller concentrations of collagen, the a.u. of the relative concentrations were rescaled by an order of 1000, such that an a.u. of 1000 equals a relative concentration of 1.

### 2.5 Collagen quantification

The average collagen concentration of each organ cross-section was then quantified within the ultrasound-segmented regions. 3D renderings of B-mode ultrasound scans with sPA collagen concentration estimates were generated using 3D Slicer [28]. We then compared the average collagen concentration between normal and prolapsed organs using a two-way analysis of variance (ANOVA) and unpaired *t*-tests. We also calculated how collagen concentration estimates in each organ along the reproductive tract correlated with age in normal and prolapsed samples using a linear regression analysis.

### 2.6 Histology calibration

We calibrated the accuracy of our spectral unmixing algorithm using ground truth collagen quantities obtained from tissue sections stained for collagen using Masson’s Trichrome, which stains collagen fibers blue. Our sPA imaging approach estimated the average concentration of collagen within cross-sectional areas of reproductive tract tissues, so our aim was to assess whether our concentration estimates aligned with histology imaging results. To obtain a comparable cross-sectional average of collagen concentration within our histological tissue sections, we calculated the fraction of area in each reproductive tract section stained blue for collagen. Reproductive organs that yielded histological sections with a higher percentage of their area occupied by collagen would therefore have higher spatial concentration of collagen. Thus, we assumed that sPA collagen concentration estimates would increase proportionally with the area fraction of collagen staining in tissue sections collected from corresponding regions.

After imaging, murine reproductive tract samples were rewashed in saline, fixed in 10 % formalin, and embedded in paraffin for sectioning. The paraffin-embedded samples were then sliced into 4-μm cross-sections every 0.5 mm along the reproductive tract to visualize the different organs. Sectioned tissues were then stained using Masson’s Trichrome Stain Kit (Epredia, Breda, Netherlands) to quantify the amount of collagen in each organ section. Whole-slide images of the stained tissues were acquired using a Zeiss Slide Scanner (Carl Zeiss AG, Jena, Germany) at 20x magnification. Each organ was identified by key anatomical features at each cross section (Fig. 2(f-i)). Fiji [29] isolated the blue-stained collagen fibers via color deconvolution. For each organ cross-section, we calculated the fraction of the tissue area that contained blue-stained pixels. Lastly, the relationship between the area fraction of collagen staining and sPA-estimated collagen concentration was quantified using a linear regression analysis.

To ensure accurate anatomical references across the reproductive tract, we included only samples that had all four organ sections visible in our dataset. Since the cervix is a very thin organ, it was difficult to obtain sections of both the internal and external os from the same sample. Therefore, histology samples were excluded from this study when missing a section of the internal os, the external os, or both. Additionally, the biomechanical properties of the *ex vivo* reproductive tract made it difficult to cleanly slice along the transverse plane, especially in prolapsed samples. Many sections had tears or out of plane folds that compromised the accuracy and integrity of the data. Thus, one normal sample and one prolapsed sample yielded complete sets of histological sections that included all four organs without any tears or out-of-plane folds.

## 3 Results

**Fig. 3:**
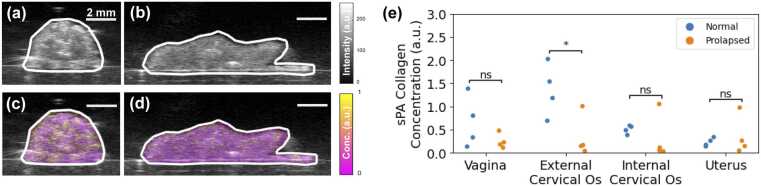
sPA imaging measures prolapse-related collagen concentration changes in the murine reproductive tract. B-mode ultrasound images of the external cervical os in (a) normal (Fbln5^+/-^) and (b) prolapsed (Fbln5^-/-^) mice both aged approximately 7.5 months. sPA collagen concentration maps of the external cervical os in age-matched (c) normal and (d) prolapsed mice. (e) sPA collagen concentration estimates in the organs along four normal and four prolapsed murine reproductive tracts aged 4–8 months. *: p < 0.05, ns: p > 0.05.

sPA imaging demonstrated that mice with prolapse aged 4–8 months had significantly less collagen (p = 2.99e-2) in the external cervical os (Fig. 3). Prolapse did not significantly affect collagen content in the internal cervical os, vagina, or uterus. Two-way ANOVA assessing mice aged 4–8 months showed that prolapse had a significant effect (p = 1.47e-2) on sPA collagen concentration estimates, while anatomical position (p = 5.64e-2) and the interaction between prolapse and anatomical position (p = 5.56e-2) did not have a significant effect. 3D renderings of sPA collagen concentration data qualitatively show differences in collagen content between normal (Video 1) and prolapsed (Video 2) reproductive tract samples from mice aged approximately 7.5 months. Figure S1 in the Supplementary Materials shows that blood oxygenation in all excised tissues was low (40 % on average) and not significantly different between normal and prolapsed groups, as was expected.

**Fig. 4:**
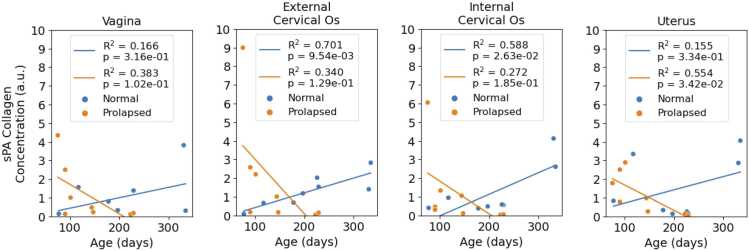
sPA imaging measures age-related collagen concentration changes in the murine reproductive tract. Linear correlations between age and sPA collagen concentration estimates in the organs along the reproductive tracts excised from eight normal mice (Fbln5^+/-^) and eight prolapsed mice (Fbln5^-/-^).

Age had a significant effect on collagen content in both *Fbln5*^*+/-*^ and *Fbln5*^*-/-*^ mice according to two-way ANOVAs assessing age with prolapse (p = 2.76e-7) and age with anatomical position (p = 3.95e-3). As shown in Fig. 4, external cervical os collagen concentration increased with age in normal mice (R^2^ = 0.701, p = 9.54e-3) and decreased with age in prolapsed mice (R^2^ = 0.340, p = 1.29e-1). While other organs did not demonstrate a clear correlation between collagen content and age, collagen content generally increased with age in normal mice and decreased with age in prolapsed mice.

**Fig. 5:**
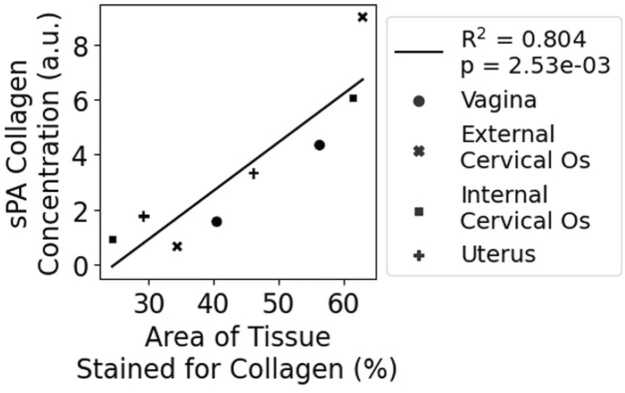
sPA collagen concentration estimations correlate with collagen staining in histological sections. Linear correlation between the fraction of tissue area stained for collagen with Masson’s trichrome and sPA collagen concentration estimates in organs across the reproductive tracts of one normal mouse (Fbln5^+/-^) and one prolapsed mouse (Fbln5^-/-^).

Histology validated a strong linear correlation (R^2^ = 0.804, p = 2.53e-3) between sPA collagen concentration estimates and Masson’s trichrome collagen staining in reproductive tissues (Fig. 5). Linear correlation included data from both *Fbln5*^*+/-*^ and *Fbln5*^*-/-*^ mice across all 3 reproductive tract organs, highlighting the robustness of the spectral unmixing algorithm.

## 4 Discussion

Our objective was to validate photoacoustic imaging as a tool for quantifying collagen content across the reproductive tract during the progression of POP. High linear correlation of the sPA estimation with histological staining confirmed the method’s reliability. With ultrasound-guided sPA we were able to observe that collagen content in murine cervixes significantly decreased with prolapse. Histological analysis confirmed the trends estimated with sPA, thereby validating ultrasound-guided sPA as a potential clinical tool for monitoring and assessing collagen content in POP cases.

Estimating collagen content with sPA holds several advantages over histological methods of collagen quantification. Histology requires a biopsy procedure to acquire the tissue sample for analysis, while PA imaging can non-invasively monitor tissue properties. Histology is also inherently time- and resource-intensive, requiring days to fix, embed, section, stain, and mount the sample before beginning image analysis. On the other hand, PA images are acquired in real time and spectral unmixing only takes a few seconds, thereby significantly reducing the time between data acquisition and analysis. Furthermore, biopsies minimize the amount of tissue excised from the patient, which limits the spatial sample size available for histological analysis. PA imaging can be repeatably applied to analyze multiple cross-sections of tissue within the same imaging session without significantly increasing the resources consumed, the time to analysis, or the risk to the patient.

The ability to quickly and non-invasively assess collagen content in reproductive tissues would also allow biomedical researchers to collect data on collagen content trends in POP patients regardless of whether they are undergoing surgery. Eliminating the need for a tissue specimen would open the possibility for longitudinal monitoring and allow clinical studies to collect data from more individuals, providing a more complete picture of how POP affects humans. Understanding how collagen behaves in prolapsed organs and their surrounding pelvic tissues may help surgeons decide which grafts to use when planning reconstructive surgeries.

Previous photoacoustic imaging research with reproductive tissues quantified collagen to water ratios in NIR-II [13], while our study focused on quantifying collagen concentration in NIR-I. Photoacoustic signals from blood are stronger in the NIR-I optical window, while water, lipid, and collagen signals are stronger in NIR-II. Because of the strong photoacoustic signals from hemoglobin in NIR-I, *in vivo* collagen concentration estimation with sPA in this wavelength range will require spectral unmixing with oxygenated and deoxygenated hemoglobin; however, this will allow for simultaneous quantification of other important health indicators such as tissue perfusion and oxygenation [10], [30]. Moreover, many commercially available PA imaging systems and conventional OPO lasers already have NIR-I imaging capabilities, meaning that reproductive tract collagen quantification in NIR-I could be implemented on existing systems at a potentially lower cost compared to quantification in NIR-II.

Future photoacoustic image processing approaches could potentially be used to analyze collagen content in specific anatomical regions. Previous studies have used collagen-dense anatomical regions such as the muscularis [17] or the subepithelium [31] of stained vaginal tissue sections as a metric to compare samples from normal and POP subjects. While collagen fiber bundles thinner than 5 µm are readily visible in some histological microscopy images [18], the spatial resolution of the LZ550 photoacoustic imaging probe used in the present study was insufficient for direct collagen fiber assessment (40 µm axial resolution, 90 µm lateral resolution). Imaging probes with higher resolution or more advanced image processing techniques are needed to replicate histological microscopy measurement methods in photoacoustic imaging analyses.

Although this study was conducted using murine reproductive tissues *ex vivo*, photoacoustic imaging shows great promise for studying collagen in human reproductive tissues *in vivo*. Previous pre-term birth research developing photoacoustic imaging methods for assessing reproductive tract collagen changes in mice *ex vivo* [13] have since been successfully demonstrated in humans *ex vivo* [14] and in mice *in vivo* [15]. Hybrid ultrasound-photoacoustic endoscopes are also currently being developed for clinical urogenital imaging applications [14], [32], [33]. Probes such as these could be used to simultaneously detect both anatomical and molecular abnormalities when screening women for POP, pre-term birth, and other reproductive health applications. *In vivo* photoacoustic imaging could be used at routine checkups to monitor longitudinal trends in reproductive tract collagen, allowing for improved risk assessment of various gynecological and obstetrical conditions.

Still, translating this *ex vivo* research to imaging applications *in vivo* will present new challenges. With larger quantities of blood and variable oxygenation *in vivo*, more signal intensity coming from hemoglobin will add to the endogenous biological noise surrounding the collagen signal. Validation experiments will need to be performed to verify the performance of the algorithm *in vivo*. Unmixing the photoacoustic signals of elastin and the different subtypes of collagen also remains challenging. Elastin is also a source of contrast in photoacoustic images but is difficult to separate from collagen via spectral unmixing because their absorption spectra are so similar [25], [34]. Dhital et al. demonstrate that reproductive tract tissues contain vastly smaller quantities of elastin compared to collagen [35]. Since collagen is generally more abundant than elastin [36], we did not consider elastin in our unmixing algorithm. It is also difficult to differentiate between the different subtypes of collagen (collagen type I, type II, type III, etc.) using sPA because they have similar molecular compositions [37]. More sophisticated algorithms will need to be developed to distinguish between the biomechanical constituents of soft tissues using sPA.

Our results showed a significant linear correlation (R^2^ = 0.701, p = 9.54e-3) between age and sPA-estimated cervical collagen content in normal, non-pregnant mice aged less than 1 year. Previous studies on normal, non-pregnant mice aged less than 1 year showed that cervical and uterine tissues became less stiff with age, but vaginal tissues became stiffer with age [19], [20], [21]. While collagen content is one factor that affects tissue stiffness, the biomechanical properties of tissues are also influenced by many other factors, including collagen alignment and cell-extracellular matrix interactions [38]. Further research is needed to clarify the relationship between photoacoustic imaging results and the biomechanical properties of reproductive tract tissues.

While this study demonstrated age-related reproductive tract collagen changes in mice, human age-related events like menopause have a significant influence on reproductive tract collagen. Menopause affects collagen in the cervix [39] and is associated with severity of prolapse [40], but mice—unlike humans—continue to cycle estrogen throughout middle age and don't have a menopause period [41]. The source of age-related collagen changes in the reproductive tract therefore differs between mice and humans. For instance, repetitive estrous cycling contributes to fibrosis in the murine uterus [42], while increased collagen in the human uterus may be attributed to inflammatory affects [43]. Although mechanisms of collagen accumulation may be different between humans and mice due to their differences in reproductive cycling with aging, this study serves as a first step in establishing ultrasound-guided sPA imaging as a method to track changes in reproductive collagen as a function of age and prolapse.

## 5 Conclusions

We developed a method to monitor changes in reproductive tract collagen content using ultrasound-guided sPA imaging in NIR-I. Collagen concentration estimation with sPA detected both prolapse-related and age-related changes in murine reproductive tract tissues. This preliminary study demonstrates the utility of sPA for characterization of tissues across the reproductive tract to detect POP. With ultrasound-guided sPA, clinicians could assess reproductive tract collagen more routinely across larger areas of the reproductive tract compared to invasive and localized tissue biopsies.

## Data Statement

Data used in this research can be made available upon request.

## CRediT authorship contribution statement

**Viraj Puri:** Writing – review & editing, Methodology, Formal analysis, Data curation, Conceptualization. **Andrew C. Markel:** Writing – review & editing, Writing – original draft, Validation, Methodology, Formal analysis, Data curation. **Bayer Carolyn L:** Writing – review & editing, Visualization, Supervision, Resources, Project administration, Methodology, Investigation, Funding acquisition, Conceptualization. **Kristin S. Miller:** Writing – review & editing, Supervision, Resources, Methodology, Investigation, Conceptualization, Funding acquisition. **Domingo Mari J. E.:** Writing – review & editing, Methodology, Conceptualization.

## Funding

This work was supported by the NSF Early Faculty CAREER Development Award CMMI-1751050, NSF CMMI 2053851, and the 10.13039/100014989Chan Zuckerberg Initiative (2021–236087).

## Declaration of Competing Interest

The authors declare that they have no known competing financial interests or personal relationships that could have appeared to influence the work reported in this paper.

## Data Availability

Data will be made available on request.

## Appendix A Supplementary material

Figure S1. Tissue oxygenation estimations derived from unmixed sPA images compared between eight normal (Fbln5 ^+/-^) and eight prolapsed (Fbln5^-/-^) murine reproductive tracts. ns: p > 0.05

Video 1. 3D rendering of ultrasound (grey) and sPA collagen concentration (yellow) data in a normal (Fbln5 ^+/-^) murine reproductive tract aged approximately 7.5 months

Video 2. 3D rendering of ultrasound (grey) and sPA collagen concentration (yellow) data in a prolapsed (Fbln5^-/-^) murine reproductive tract aged approximately 7.5 months
