# Supplementary material for: Spectral photoacoustic imaging of age-related reproductive tract collagen changes in a mouse model of prolapse
Source: Photoacoustics. 2026 Feb 12;48:100810. doi: 10.1016/j.pacs.2026.100810 (PMC12926569; doi:10.1016/j.pacs.2026.100810)
Supplement: Supplementary file 1 — Figure S1. Tissue oxygenation estimations derived from unmixed sPA images compared between eight normal (Fbln5 +/-) and eight prolapsed (Fbln5-/-) murine reproductive tracts. ns: p > 0.05 [file mmc1.docx]

**Supplementary Materials for:**

**Spectral photoacoustic imaging of age-related reproductive tract collagen changes in a mouse model of prolapse**

Andrew C. Markel^1^, Viraj Puri^1^, Mari J. E. Domingo^1^, Kristin S. Miller^1,2,3^, Carolyn L. Bayer^1^

^1^Department of Biomedical Engineering, Tulane University, 500 Lindy Boggs Center, New Orleans, LA 70118, USA

^2^Department of Bioengineering and Mechanical Engineering, University of Texas at Dallas, 800 W. Campbell Road, Richardson, TX, 75080, USA

^3^Department of Obstetrics and Gynecology, University of Texas Southwestern Medical Center, 5323 Harry Hines Blvd, Dallas, TX, 75390, USA


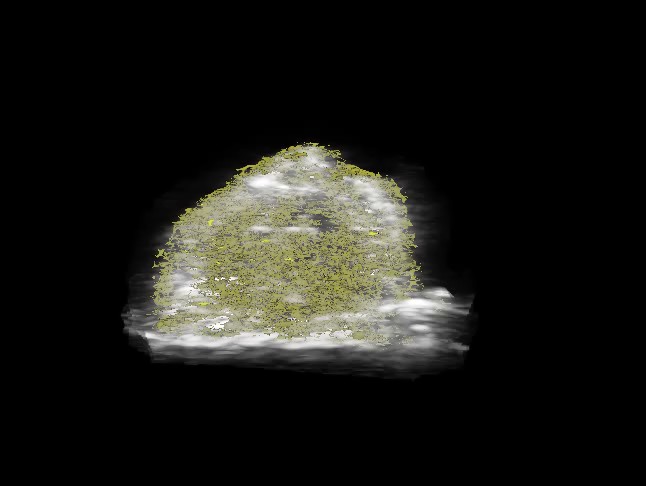


(Still used to reference video)

Video 1. 3D rendering of ultrasound (grey) and sPA collagen concentration (yellow) data in a normal (Fbln5+/-) murine reproductive tract aged approximately 7.5 months.


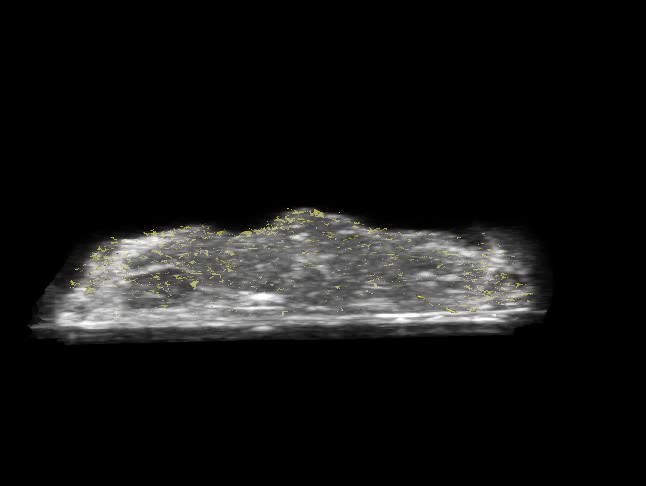


(Still used to reference video)

Video 2. 3D rendering of ultrasound (grey) and sPA collagen concentration (yellow) data in a prolapsed (Fbln5-/-) murine reproductive tract aged approximately 7.5 months.


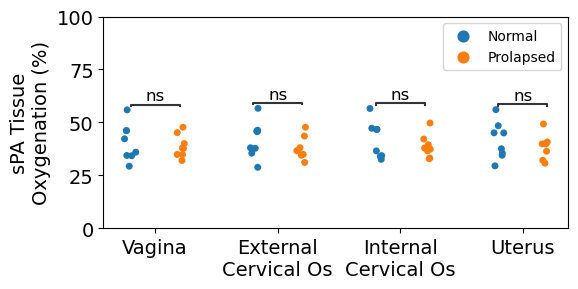


Figure S1. Tissue oxygenation estimations derived from unmixed sPA images compared between eight normal (Fbln5+/-) and eight prolapsed (Fbln5-/-) murine reproductive tracts. ns : p > 0.05.
